# An Electron Paramagnetic Resonance Study of the Superoxide-Scavenging and Redox-Modulating Effects of Lecithinized Superoxide Dismutase in the Bloodstream

**DOI:** 10.3390/molecules30091882

**Published:** 2025-04-23

**Authors:** Dessislava Lazarova, Plamen Getsov, Rumiana Bakalova, Biliana Nikolova, Severina Semkova, Zhivko Zhelev, Zhiwei Qiao, Tomohiro Ishikawa, Koichiro Fukuda, Kensuke Osada, Milka Mileva, Tohru Mizushima, Ichio Aoki

**Affiliations:** 1 Faculty of Medicine, Sofia University “St. Kliment Ohridski”, 1407 Sofia, Bulgaria; 2 Faculty of Medicine, Medical University, 1000 Sofia, Bulgaria; 3 Institute of Biophysics and Biomedical Engineering, Bulgarian Academy of Sciences, 1113 Sofia, Bulgaria; 4 Faculty of Medicine, Trakia University, 6000 Stara Zagora, Bulgaria; 5 LTT Bio-Pharma Co., Ltd., Tokyo 105-0013, Japan; qiao@ltt.co.jp (Z.Q.); t.ishikawa@ltt.co.jp (T.I.); fukuda@ltt.co.jp (K.F.);; 6 Institute for Quantum Medical Science, National Institutes for Quantum Science and Technology (QST), Chiba 263-8555, Japan; 7 Stephan Angeloff Institute of Microbiology, Bulgarian Academy of Sciences, 1113 Sofia, Bulgaria

**Keywords:** redox state, inflammation, oxidative stress, lecithinized superoxide dismutase, EPR spectroscopy

## Abstract

Lecithinized superoxide dismutase (PC-SOD) was found to have a significantly improved half-life in the bloodstream and better pharmacological effects compared with unmodified SOD. However, there is no direct evidence that parenterally administered PC-SOD decreases superoxide levels in blood and tissues in vivo. In the present study, we investigated the ability of PC-SOD versus unmodified SOD as a superoxide scavenger in mice subjected to oxidative stress. Experiments were performed on a lipopolysaccharide (LPS) mouse model of acute inflammation known to be accompanied by the overproduction of superoxide in the blood. The mice were divided into four groups: untreated (healthy; *n* = 6), LPS-treated (*n* = 7), LPS/SOD-treated (*n* = 6), and LPS/PC-SOD-treated (*n* = 7) mice. SOD and PC-SOD were injected intravenously. Blood samples were collected at four time intervals and analyzed by electron paramagnetic resonance (EPR) spectroscopy using a nitroxide probe, 3-carbamoyl-2,2,5,5-tetramethylpyrrolidine-1-oxyl (CMP). The following effects were observed: (i) In the blood of healthy mice, the EPR signal was significantly lower compared with the control (*p* < 0.001) and LPS-treated mice (*p* < 0.01); (ii) in the blood of LPS-treated mice, the EPR signal was identical to that of the control; and (iii) in the blood of LPS/SOD-treated mice collected immediately after enzyme injection, the EPR signal was significantly lower compared with the control (*p* < 0.01) and LPS-treated mice (*p* < 0.05). However, the effect disappeared in the samples collected 30 min and 1 h after enzyme injection. (iv) In LPS/PC-SOD-treated mice, the EPR signal was significantly lower compared with the control (*p* < 0.01) and LPS-treated mice (*p* < 0.05), even in the blood samples collected within 1 h after enzyme injection. The data indicate that the blood of healthy mice was characterized by a high reducing capacity, while the blood of LPS-treated mice was characterized by a high oxidative capacity. SOD decreased superoxide production immediately after enzyme injection. However, the effect was short-lived and disappeared within 30 min. PC-SOD effectively decreased superoxide production in the bloodstream of LPS-treated mice and restored the redox balance to the control level even two hours after enzyme injection. The effects of PC-SOD were more pronounced and long-lasting compared with those of SOD. The possible reason is the longer half-life of PC-SOD in the bloodstream, its better stability, and its slower clearance from the circulation due to the increased hydrophobicity of the enzyme and its interaction with plasma proteins. The data are discussed in the context of recent clinical trials showing that PC-SOD is a promising pharmaceutical product for adjuvant therapy of a variety of pathologies accompanied by inflammation, redox imbalance, and oxidative stress.

## 1. Introduction

Reactive oxygen species (ROS) are considered a major inducer of redox imbalance and oxidative stress in mammals [1,2,3]. However, ROS are not just a harmful byproduct of cellular function. They are important signaling molecules and regulators of many biochemical and physiological processes, from metabolism to the immune response [2,3]. The most important ROS that have been shown to be directly or indirectly involved in cell signaling are superoxide (O_2_^•−^), hydrogen peroxide (H_2_O_2_), and nitric oxide (NO). They are an indispensable part of cellular biochemistry, produced endogenously in normal amounts, and their pathogenic effects are manifested at over-threshold concentrations. The main factor for maintaining ROS within physiological limits is endogenous reducing equivalents: enzymatic antioxidants [superoxide dismutase (SOD), catalase, glutathione peroxidase, etc.], non-enzymatic antioxidant systems, thiol-containing proteins (thioredoxin, peroxiredoxin, and glutaredoxin), and some cofactors (NADH and NADPH).

ROS and reducing equivalents are often described as “redox-active compounds” and the balance between them as the “redox state” (redox status) or “bioreductive capacity” of cells, tissues, and body fluids [4,5]. The redox state mediates the relationship between the replication, transcription, and translation of genetic information [6], as well as the organization of intermolecular and metabolic interactions in living organisms [7]. Redox imbalance plays an important role in aging and “free radical pathologies” accompanied by chronic or acute inflammation, such as viral, bacterial, and parasitic infections; malignant, autoimmune, atherogenic, and neurodegenerative diseases; type 2 diabetes; and others [1,2,3,8,9,10]. Therefore, the redox state is currently considered one of the most valuable diagnostic markers and therapeutic targets. Efforts are aimed at developing new drugs and methodologies to regulate this parameter in cells, tissues, and body fluids and prevent oxidative stress in organisms.

SOD is a first-line defense against oxidative stress and one of the main enzymatic endogenous regulators of the redox state in living biological objects. The enzyme eliminates excess superoxide radicals generated under physiological conditions, maintaining their level within normal limits necessary for the regulation of cell signaling. It also eliminates superoxide radicals in pathological conditions, which leads to the production of large amounts of highly cytotoxic hydrogen peroxide, inducing apoptosis and cell death. SOD has proven to be a powerful tool against “free radical diseases”, despite some limitations in the therapeutic effects of its native unmodified isoforms [11,12]. Chemically modified forms of SOD and SOD mimetics are being developed to enhance antioxidant and anti-inflammatory effects and improve therapeutic efficacy, which has been demonstrated in multiple animal models as well as in human clinical trials summarized in recent review articles [11,13].

Our study aimed to investigate the superoxide-scavenging and redox-modulating effects of a chemically modified (lecithinized) superoxide dismutase (PC-SOD). PC-SOD is a recombinant human cytosolic Cu,Zn-SOD conjugated with four molecules of phosphatidylcholine. This chemical modification was found to increase the hydrophobicity of the enzyme, which interferes with its pharmacokinetics, bioavailability in the bloodstream, and biodistribution into the cells and tissues [14,15,16].

The lecithinized enzyme has been shown to overcome the clinical limitations of unmodified SOD and exhibit beneficial anti-inflammatory effects in animal models of acute respiratory distress syndrome and ventilator-induced lung injury [14]. Several clinical trials have demonstrated its safety and efficacy in humans with intravenous administration [14,15,16,17]. To our knowledge, there is no direct evidence that parenterally administered PC-SOD decreases superoxide levels in the blood, as well as no studies proving the superoxide-scavenging effects of PC-SOD compared with native SOD in vivo.

**Figure 1:**
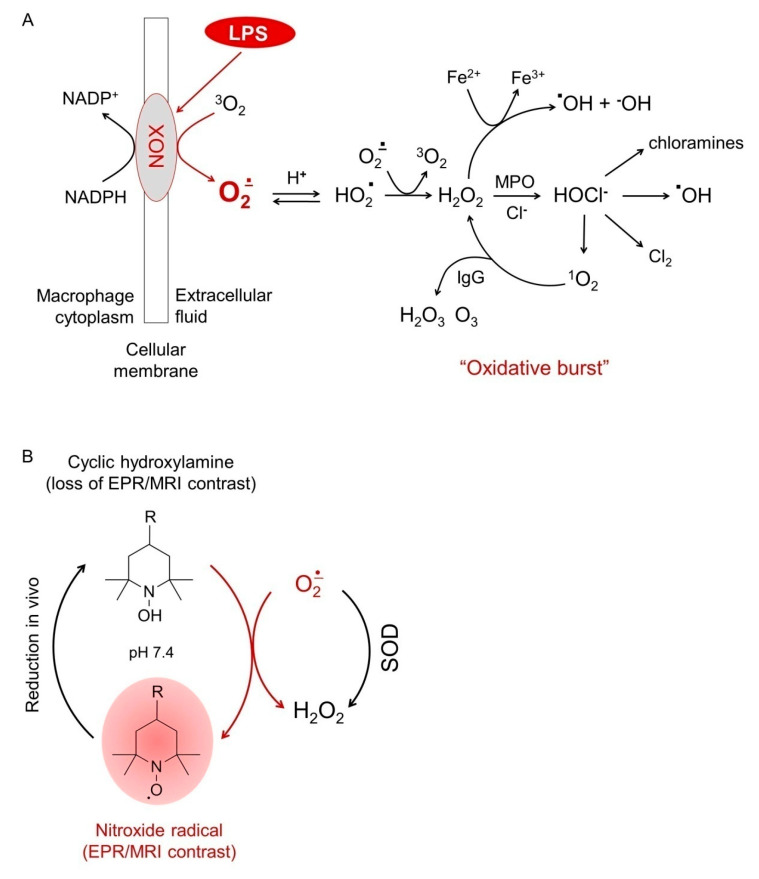
(**A**) Induction of “oxidative burst” in lipopolysaccharide (LPS)–activated macrophages: molecular mechanism. LPS up–regulates and activates NADPH–dependent oxidase complex (NOX), located on the cellular membranes of macrophages [18,19,20,21]. The process is accompanied by the overproduction of superoxide as a primary product in the extracellular environment. This triggers a cascade of reactions, leading to the production of various reactive oxygen species, called an “oxidative burst”. (**B**) The redox cycle of a nitroxide derivative, the dynamics of its EPR/MRI signal intensity, and the effects of superoxide and superoxide dismutase (SOD) on this process. In vivo, in norm, the paramagnetic nitroxide radical reacts with various reducers to form the diamagnetic hydroxylamine. Superoxide oxidizes hydroxylamine to the nitroxide radical and restores its EPR/MRI contrast. Superoxide dismutase (SOD) competes with hydroxylamine for superoxide. This interferes with the conversion of hydroxylamine to the nitroxide radical and the EPR/MRI signal, respectively. EPR—electron paramagnetic resonance; IgG—immunoglobulin G; MPO—myeloperoxidase; MRI—magnetic resonance imaging.

The lipopolysaccharide (LPS) mouse model of inflammation is one of the most suitable and widely used in vivo models for studying the superoxide-scavenging and redox-modulating effects of drugs and natural substances, including SOD and SOD mimetics. LPS is a generic term of serological toxins isolated from the bacterial wall and is the most popular exogenous inducer of ROS in an organism. Its mechanism of action is directly related to the induction of an “oxidative burst” of macrophages via activating the NADPH-dependent oxidase complex (NOX) located on their cell membranes [18,19] (Figure 1A). The process involves the generation of a huge amount of superoxide as a primary product in the extracellular environment [18,19]. It has recently been reported that LPS up-regulates the NOX1 isoenzyme, which is also accompanied by the overproduction of superoxide as well as cytokines [20,21].

In the last decade, significant progress has been made in the selective localized detection of redox-active compounds in vitro and in vivo [4,5,22]. Nitroxide-enhanced magnetic resonance techniques such as EPR and MRI are some of the most sensitive and specific methods for visualizing and analyzing superoxide in biological objects, as well as for the assessment of redox imbalance and oxidative stress, especially in vivo/in situ, induced by various factors, including LPS [23]. Furthermore, EPR imaging using cyclic nitroxides as redox-sensitive probes allows the direct measurement of superoxide production with high selectivity and sensitivity, which is difficult to achieve by other imaging techniques, such as fluorescent, chemiluminescent, spectrophotometric, and others.

Cyclic nitroxides exist in three forms: paramagnetic long-lived nitroxide radical, diamagnetic hydroxylamine, and diamagnetic oxoammonium (Figure 1B). Nitroxide radical participates in electron transfer reactions with numerous endogenous redox-active substances (oxidizers and reducers), with the formation of non-contrast diamagnetic intermediates [23,24,25]. The rate constants of these reactions determine the intensity and dynamics of the nitroxide-enhanced EPR/MRI signal in cells, tissues, and body fluids. It is generally accepted that in vivo cyclic nitroxides exist mainly in two forms, nitroxide radical and hydroxylamine, due to the rapid reduction of oxoammonium to hydroxylamine by NAD(P)H at physiological pH [23,24,25]. Hydroxylamine is a SOD mimetic and can restore the nitroxide radical and its EPR/MRI signal by efficiently interacting with superoxide. Therefore, in living biological objects, various redox-active substances can participate directly or indirectly in the formation of oxoammonium and hydroxylamine and lead to the loss of the EPR/MRI signal, but only the interaction of hydroxylamine with superoxide can restore the radical form of nitroxide and its EPR/MRI contrast. Thus, the dynamics of the EPR/MRI signal of nitroxide radicals can serve as a marker for assessing the redox imbalance and the level of oxidative stress in cells, tissues, and body fluids caused by the overproduction of superoxide. This makes nitroxide-enhanced EPR/MRI imaging a suitable technique to study the direct superoxide-scavenging effects of SOD and its chemically modified forms, such as PC-SOD.

Based on the facts described above, our study aimed to elucidate the superoxide-scavenging and redox-modulating effects of PC-SOD compared with unmodified SOD administered intravenously in LPS-treated mice using EPR spectroscopy and 3-carbamoyl-2,2,5,5-tetramethylpyrrolidine-1-oxyl (CMP) as a redox-sensitive probe. CMP belongs to the group of hydrophilic pyrrolidine nitroxides with very slow penetration into living cells [23,24], making the probe suitable to study the redox state of body fluids. CMP is highly selective for superoxide but also interacts with multiple reducers, which allows the assessment of both the superoxide-scavenging and redox-modulating effects of SOD and PC-SOD in the circulation. It should be noted that cyclic hydroxylamine also undergoes nonspecific oxidation, for example, by ambient oxygen, but the rate of this process has been found to be negligibly low compared with the rate of hydroxylamine oxidation by superoxide [24,25]. It has also been reported that the reaction of cyclic hydroxylamines with superoxide is relatively fast, two orders of magnitude faster than spin trapping, and the nitroxide radicals formed are more stable than spin trapping adducts [24,25]. This allows using nitroxide probes in small and harmless concentrations [24]. The above observations determined the choice of CMP as a redox-sensitive probe in our study. To our knowledge, this is the first EPR study that provides direct evidence for the superoxide-scavenging effects of PC-SOD and SOD in the blood after intravenous administration of both enzymes in mice with LPS-induced inflammation. This study also showed that the redox-modulating effects of PC-SOD in the blood were longer-lasting compared with SOD.

## 2. Results

The first step of our study was to select the appropriate conditions for the overproduction of superoxide and the induction of redox imbalance in the blood of mice by LPS-induced inflammation, as well as to select the optimal concentration of SOD that effectively suppresses superoxide production and oxidative stress in the blood and normalizes the redox state without severe side-effects. For this purpose, we used two concentrations of LPS (i.p.-injected)—1 mg and 10 mg per kg b.w.—based on the already published data from “LPS mouse models of inflammation” [14,20,26]. The design of the treatment protocol was based on studies demonstrating that LPS at the selected doses induced inflammation within 2–4 h after i.p. injection through macrophage activation and the overproduction of superoxide as the primary product of the NOX-mediated “oxidative burst” [18,19,20,21]. The higher concentration of LPS caused a rapid acute inflammatory response in the mice, characterized by an increased temperature (38–39 °C), sometimes accompanied by fever. Under such conditions, the uncontrolled overproduction of various types of ROS and severe oxidative stress compromised EPR analyses, most likely due to the conversion of nitroxide radical predominantly to diamagnetic oxoammonium [23,24,27] but not to hydroxylamine due to the crash of antioxidant and reducing systems. For this reason, we preferred to treat the animals under more sparing conditions, i.e., 1 mg of LPS per kg b.w. The mice developed a subfebrile temperature (about 37.5 °C) within two hours after LPS i.p. injection, indicating moderate inflammation. In this case, the dynamics of the EPR signal of the nitroxide probe in the blood were approximately the same and predictable in all LPS-treated animals.

In previous studies, it was established that PC-SOD at doses of 1 mg/kg and 3 mg/kg suppressed the anti-inflammatory effects of LPS, decreasing the production of pro-inflammatory cytokines such as IL-6 and TNF-alpha, with the effects being more pronounced at a higher dose of the enzyme [14,28,29]. The effects are less pronounced above 3 mg of PC-SOD per kg b.w. [29]. In a pilot experiment, we also found that PC-SOD i.v.-administered at doses of 6 and 8 mg/kg b.w. induced systemic toxicity in mice and decreased their survival.

**Figure 2:**
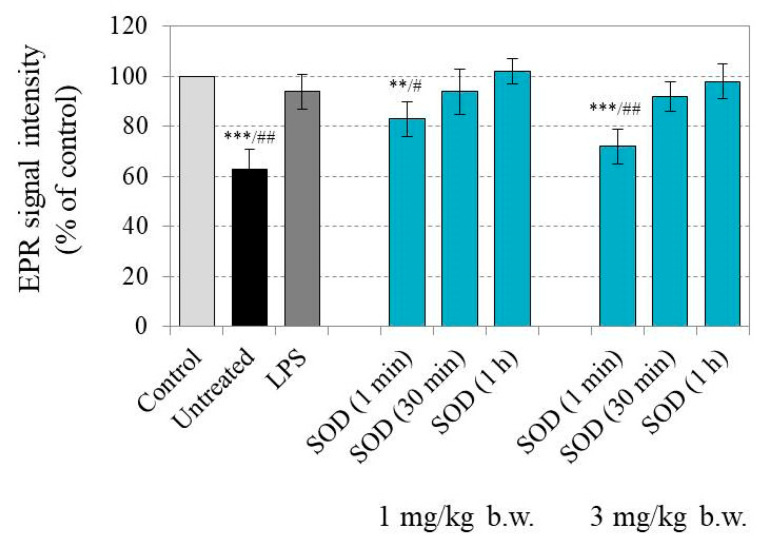
EPR signal intensity, recorded after addition of the CMP (1 mM) to the corresponding blood sample isolated from healthy (untreated), LPS-treated, and LPS/SOD-treated mice. Blood samples were isolated immediately (1 min), 30 min, and 1 h after i.v. injection of SOD (1 mg/kg b.w. or 3 mg/kg b.w.). CMP was incubated with blood for 15 min before EPR measurement. The EPR signal intensity of CMP (1 mM) in the buffer, in the absence of biological material, was taken as the control and considered 100%. All results were calculated as a percentage of this control. The data are presented as means ± SD from two independent EPR measurements for each blood sample collected from different numbers of mice in the group: untreated (*n* = 6), LPS-treated (*n* = 7), and LPS/SOD-treated (*n* = 6 in both subgroups at each blood sampling interval). *n*—number of mice. ** *p* < 0.01, and *** *p* < 0.001 versus control; ^#^ *p* < 0.05, ^##^ *p* < 0.01 versus LPS-treated group.

Figure 2 shows the EPR signal of CMP in blood samples isolated from untreated (healthy), LPS-treated, and LPS/SOD-treated mice. The EPR signal of CMP in the buffer, in the absence of biological material, was taken as the control and considered 100%. Blood was collected from the mice and separated into two aliquots. CMP was added to each blood aliquot and analyzed by EPR spectroscopy at 15 min of incubation. In untreated mice, the EPR signal decreased significantly without disappearing completely. These data indicated the partial reduction of the contrast nitroxide radical to the non-contrast hydroxylamine in the bloodstream. In LPS-treated mice, the intensity of the EPR signal was similar to the control and constant at 15 min of incubation of CMP with blood. These data show that CMP remained in its radical form, which is an indication of the presence of higher amounts of superoxide and an increased oxidizing capacity in the blood of LPS-treated mice compared with untreated mice.

SOD was injected into the LPS-treated mice in two doses: 1 mg/kg b.w. and 3 mg/kg b.w. Blood was collected immediately (1 min), 30 min, and 1 h after SOD injection, and each blood sample was separated into two aliquots. CMP was added to each aliquot, and the EPR signal was recorded at 15 min of incubation. At both enzyme doses, the EPR signal in the blood isolated immediately after SOD injection decreased significantly compared with the control sample and the LPS-treated group, while the EPR signal in blood isolated 30 min and 1 h after SOD injection did not change compared with the control, as well as compared with the LPS-treated group. This shows that SOD suppressed superoxide production and restored the reducing capacity of the blood of LPS-treated mice. However, the effect was short-lived, disappearing 30 min after the injection of the enzyme. The effect of the higher dose of SOD (3 mg/kg b.w.) on the reducing capacity of the blood of LPS-treated mice was more pronounced. In this case, the EPR signal of CMP in the blood, isolated immediately after SOD injection, was similar to that of untreated (healthy) mice, and the change was statistically significant compared with mice treated with LPS and 1 mg of SOD per kg b.w. (*p* < 0.05) (Figure 2).

In further experiments, we compared the superoxide-scavenging effects of 3 mg/kg b.w. of SOD with that of PC-SOD in the blood of LPS-treated mice.

**Figure 3:**
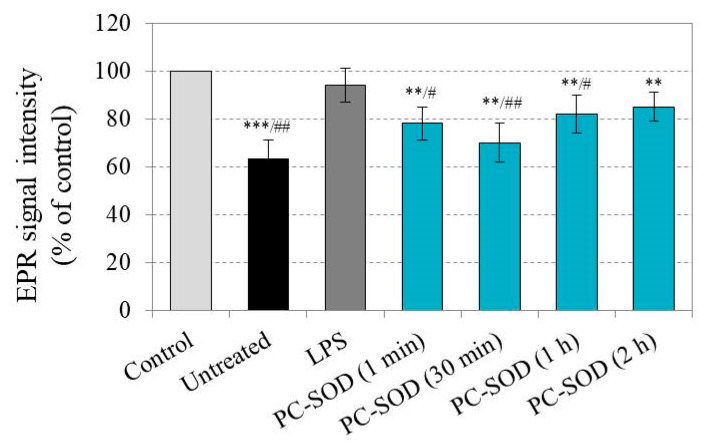
EPR signal intensity, recorded after addition of the CMP (1 mM) to the corresponding blood sample isolated from healthy (untreated), LPS-treated, and LPS/PC-SOD-treated mice. Blood samples were isolated immediately (1 min), 30 min, 1 h, and 2 h after i.v. injection of PC-SOD (3 mg/kg b.w.). CMP was incubated with blood for 15 min before EPR measurement. The EPR signal intensity of CMP (1 mM) in the buffer, in the absence of biological material, was taken as the control and considered 100%. All results were calculated as a percentage of this control. The data are presented as means ± SD from two independent EPR measurements for each blood sample collected from different numbers of mice in the group: untreated (*n* = 6), LPS-treated (*n* = 7), and LPS/C-SOD-treated (*n* = 7 at each blood sampling interval). *n*—number of mice. ** *p* < 0.01, and *** *p* < 0.001 versus control; ^#^ *p* < 0.05, ^##^ *p* < 0.01 versus LPS-treated group.

The intensity of the EPR signal of CMP in blood samples, isolated from LPS/PC-SOD-treated mice, is shown in Figure 3. Blood was collected immediately (1 min), 30 min, 1 h, and 2 h after PC-SOD injection (3 mg/kg b.w.), and each blood sample was separated into two aliquots. CMP was added to each aliquot, and the EPR signal was recorded at 15 min of incubation. In all blood samples, the EPR signal significantly decreased, with the effect being more pronounced in blood samples isolated 30 min after PC-SOD injection. This shows that PC-SOD suppresses superoxide production and restores the reducing capacity of the blood of LPS-treated mice. The effect of PC-SOD was longer lasting than that of SOD (Figure 3 versus Figure 2). PC-SOD substantially decreased the EPR signal of CMP, indicating an effective suppression of superoxide production when the samples were collected even at 2 h after enzyme injection.

It should be noted that SOD and PC-SOD did not significantly affect the EPR signal intensity in blood samples isolated from healthy mice. This was found in blood isolated from three animals in each group (immediately and 30 min after enzyme injection).

## 3. Discussion

Over the past two decades, several EPR studies have described the effects of SOD and its modified forms on the superoxide level and redox state of cells, tissues, and body fluids in LPS models of chronic and acute inflammation [30,31,32,33].

Dikalov et al. (2002) used the negatively charged cyclic hydroxylamine 1-hydroxy-4-phosphonooxy-2,2,6,6-tetramethylpiperidine (PP-H) and EPR spectroscopy to detect superoxide production in LPS-treated cultured macrophages as well as in the blood of LPS-treated rats [30]. The authors found that PP-H reacts with superoxide in LPS-treated objects, generating the stable nitroxide radical 4-phosphonooxy-TEMPO. In LPS-treated macrophages, a three-fold increase in the superoxide level was observed, which was confirmed by the appearance of a clearly expressed EPR signal and its increase with the time of incubation of the cells with LPS. SOD suppressed the appearance of the EPR signal in LPS-treated cells to the level recorded in untreated cells. A distinct EPR signal of 4-phosphonooxy-TEMPO was also recorded in the blood of LPS-treated rats. In this case, the superoxide production increased in a dose-dependent manner: from 2.2-fold at 1 mg of LPS per kg to 4.5-fold at 10 mg of LPS per kg. Blood samples were analyzed 5 h after LPS injection. The effects of SOD have not been studied in an in vivo model.

Elajalli et al. (2019) used EPR spectroscopy and two nitroxide probes to analyze cellular and mitochondrial superoxide production in mice i.p.-treated with LPS (10 mg/kg b.w.) for 24 h, inducing lung injury [33]. The authors analyzed three types of mice: wild type; lacking total body extracellular SOD (EC-SOD KO); and overexpressing lung extracellular SOD (EC-SOD Tg). The EPR data showed that the cellular and mitochondrial superoxide level was increased in the lungs of LPS-treated mice compared with controls. Cellular superoxide was increased in the lungs of EC-SOD KO mice and decreased in EC-SOD Tg mice compared with wild-type mice.

Okazaki et al. (2014) used a “sepsis model” of acute oxidative stress induced in mice by i.p. injection of a high dose of LPS (150 mg/kg b.w.) to analyze the effects of i.v.-injected pegylated SOD (PEG-SOD) and catalase in combination on the redox state of tissues and blood [32]. The authors administered intravenously 1-acetoxy-3-carbamoyl-2,2,5,5-tertamethylpyrrolidine (ACP) as a redox-sensitive probe and used EPR imaging of the whole body and circulating blood. Images were obtained on intact mice within 40 min after ACP injection. ACP is a diamagnetic precursor of CMP. ACP is hydrolyzed in mice to cyclic nitroxide CMP, which exists in two forms: paramagnetic (radical) and diamagnetic (hydroxylamine) forms [32,34]. The authors reported that the in vivo EPR signal increased up to 7–8 min after the ACP injection due to its conversion into paramagnetic CMP radicals and then decreased within 40 min due to the partial reduction of CMP radicals to hydroxylamine, as well as the clearance of the nitroxide probe from the organism. The decay of the EPR signal in the tissues of LPS-treated mice was significantly slower than that in healthy (untreated) mice, although the rate of clearance of the nitroxide probe was the same in both groups. In their study, no significant difference was observed in the dynamics of EPR signal decay in vivo in the blood of healthy and LPS-treated mice. In our preliminary experiments, we also found that in a model of acute inflammation in mice induced by a high dose of LPS (10 mg/kg b.w.), the dynamics of the EPR signal of CMP in the blood was unstable. In some specimens, the signal increased, and in others, it decreased, compared with that in healthy mice, and the effect was not repeatable. We hypothesize that this may be due to the uncontrolled production of large amounts of various ROS, including hydroxyl radicals, by macrophages in the blood at high doses of LPS, as it is in a “sepsis model” (Figure 1A). Superoxide oxidizes hydroxylamine to nitroxide radicals, which is accompanied by an increase in the EPR signal. However, hydroxyl radicals oxidize nitroxide radicals to diamagnetic oxoammonium, which is accompanied by a decrease in the EPR signal. The ratio between the speed of these two processes determines the dynamics of the EPR signal and its high variability. Furthermore, their experimental protocol differed significantly from ours, which may also be one of the reasons for the observed differences in the effect of LPS on the EPR signal in blood. Okazaki et al. (2014) also found that the suppression of whole-body EPR signal decay in LPS-treated mice was abolished by PEG-SOD and catalase, as well as by nitric oxide synthase inhibitors, whereas the enzymes did not significantly affect EPR signal dynamics in healthy mice [32]. Their article did not mention the dose of the injected enzymes. Based on these observations, the authors consider that superoxide and peroxynitrite, produced in the “oxidative burst” of macrophages, are responsible for LPS-induced oxidative stress in mice.

Pandian et al. (2005) reported increased superoxide production in isolated LPS-treated mouse aortic endothelial cells, as analyzed by EPR spectroscopy [31]. The authors demonstrated that LPS-induced superoxide production was inhibited by diphenyleneiodonium (DPI) and stimulated by NADPH, suggesting the Involvement of NOX in the process. 5,5-dimethyl-1-pyrroline-N-oxide (DMPO) was used as a spin-trapping reagent. Quantification of the spectrum of the DMPO-OH adduct, which is a result of the interaction between DMPO and superoxide, was performed by the simulated fitting of the experimental data with that of the nitroxide radical TEMPOL, measured under identical conditions. The authors used SOD to confirm the origin of the DMPO-OH adduct. The article stated that the enzyme caused complete inhibition of the characteristic quartet EPR spectrum, although these data were not presented in the figures. Nevertheless, their data showed significant superoxide production in LPS-treated mice, which was inhibited by SOD in isolated aortic endothelial cells.

A pharmaceutical form of SOD (orgotein) has been known since the 1970s as a safe anti-inflammatory agent. Orgotein for intra-articular injection has been used to manage the treatment of osteoarthritis for many years in Europe [35]. A placebo-controlled efficacy, safety, and dose comparison study in patients receiving 8–32 mg (once weekly) for three weeks reported that the long-term effects of intra-articular administration of SOD contributed to a favorable risk–benefit ratio [35]. This clinical trial also supports the opinion that superoxide is crucial in the pathology of osteoarthritis.

Recent clinical trials have reported the safety, tolerability, and effectiveness of PC-SOD administered intravenously in patients with ulcerative colitis [16], idiopathic interstitial pneumonia [36], and peripheral neuropathy [37]. In patients with ulcer colitis, PC-SOD was injected once daily in doses of 40 mg and 80 mg [16]. At 4 weeks of PC-SOD treatment, the disease activity index (DAI) decreased significantly from baseline in both groups. In patients with progressive idiopathic interstitial pneumonia, a double-blind controlled trial evaluated the safety and tolerability of PC-SOD when administered at doses of 40 mg and 80 mg [36]. PC-SOD has been reported to be safe and improves the levels of serum markers such as lactate dehydrogenase and surfactant protein-A in these patients. A double-blind randomized study evaluated the efficacy of PC-SOD at a dose of 80 mg to prevent peripheral neuropathy in patients with colorectal cancer treated with oxaliplatin [37]. Treatment with PC-SOD has been reported to suppress peripheral neuropathy, which limits the application of oxaliplatin in cancer treatment.

The studies discussed above have shown that chemically modified forms of SOD, such as PC-SOD and PEG-SOD, have better pharmacological and therapeutic effects compared with the unmodified enzyme. In the case of PC-SOD, the insertion of lecithin residues into the SOD molecule increases the hydrophobicity of the enzyme and its binding to serum protein(s) such as albumin and plasma membrane microdomains, whereas unmodified SOD does not [28]. These effects enhance the stability of PC-SOD in the bloodstream. It was also reported that PC-SOD is distributed in plasma but not in blood cells after addition to blood, and the modified enzyme circulates longer in the bloodstream of mice compared with unmodified SOD [28]. The long half-life and high plasma concentration of PC-SOD upon i.v. administration has also been confirmed in humans in a phase I clinical trial for safety [38]. PC-SOD is easily delivered from the blood to tissues by entering cells via cholesterol-sensitive endocytosis [39]. Thus, PC-SOD was found to have a significantly longer life in the circulation, as well as better biodistribution and pharmacological effects compared with unmodified SOD [15,16]. This explains the longer-lasting effects of superoxide scavenging and redox modulation in the blood of LPS-treated mice compared with the unmodified enzyme observed in our study. PEG-SOD also shows increased stability and prolonged circulation compared with SOD due to the increased molecular weight of the PEGylated enzyme and reduced renal filtration [40,41]. In addition, PEGylation decreases the immunogenicity of the enzyme and improves its therapeutic efficacy in oxidative stress models [40].

It should be noted that our study suffered from some limitations, such as the use of a single dose of PC-SOD and the lack of a stability test of the enzyme in the blood after its i.v. injection into mice. For the stability of PC-SOD in blood, we proceeded from the published studies discussed above. The choice of dose was based on our preliminary experiments, indicating that PC-SOD did not exhibit adverse side effects, such as systemic toxicity and lethality, at doses of up to 3 mg/kg in wild-type mice. The safety and tolerability of the enzyme have been confirmed in clinical studies, with doses of up to 160 mg/day [15,16,38], which corresponds to ~2 mg/kg body weight per day for adults. Our future efforts will be directed toward a systematic study to overcome the aforementioned limitations, as well as to clarify the role of PC-SOD in the key molecular mechanisms of redox regulation in an organism.

## 4. Materials and Methods

### 4.1. Chemicals

3-carbamoyl-2,2,5,5-tetramethylpyrrolidine-1-oxy (cat. #155683), LPS from *Escherichia coli* (cat. #L2630), and SOD from bovine erythrocytes (cat. #S7571) were purchased from Sigma-Aldrich. PC-SOD was prepared by LTT Bio-Pharma Co., Ltd. (Tokyo, Japan) using the same isoenzyme form.

Deionized water (deionization by the Milli-Q system) was used for all experiments. The other chemicals used were of analytical or HPLC grade.

### 4.2. Animals

C57Bl/6 mice (wild type; male; 6 to 8 weeks of age at the time of the experiments; mean weight: ~25 g) were used. All experiments were conducted in accordance with the guidelines of Bulgaria’s Directorate of Health Prevention and Humane Behavior toward Animals and approved by the Animal Care and Use Committee of the Stephan Angeloff Institute of Microbiology with protocol no. 25 from 8 May 2020 for a five-year period. The number of mice approved for this study was 55.

The mice were housed in standard cages with a wire-bar lid, at room temperature ~22°C, and a 12:12 light/dark cycle (200 lx). The maximum number of animals per cage was five. The diet was standard. The mice were separated into the following groups: (i) untreated (healthy) (*n* = 6); (ii) LPS-treated (1 mg/kg body weight (b.w.) (*n* = 7)); (iii) LPS-treated (10 mg/kg b.w.) (*n* = 5); (iv) LPS/SOD-treated (LPS: 1 mg/kg b.w.; SOD: 1 mg/kg b.w.) (*n* = 6 at each blood sampling interval); (v) LPS/SOD-treated (LPS: 1 mg/kg b.w.; SOD: 3 mg/kg b.w.) (*n* = 6 at each blood sampling interval); (vi) LPS/PC-SOD-treated (LPS: 1 mg/kg b.w.; SOD: 3 mg/kg b.w.) (*n* = 7 at each blood sampling interval); (vii) PC-SOD-treated (3 mg/kg) (*n* = 3); and (viii) SOD-treated (3 mg/kg) (*n* = 3).

LPS was injected intraperitoneally (i.p.) in a 50 μL sample volume. SOD and PC-SOD were injected intravenously (i.v.) in a 100 μL sample volume.

The mice were euthanized using pentobarbital (150 mg/kg body weight) by cervical dislocation under 4% isoflurane anesthesia.

### 4.3. Experimental Protocol

**Figure 4:**
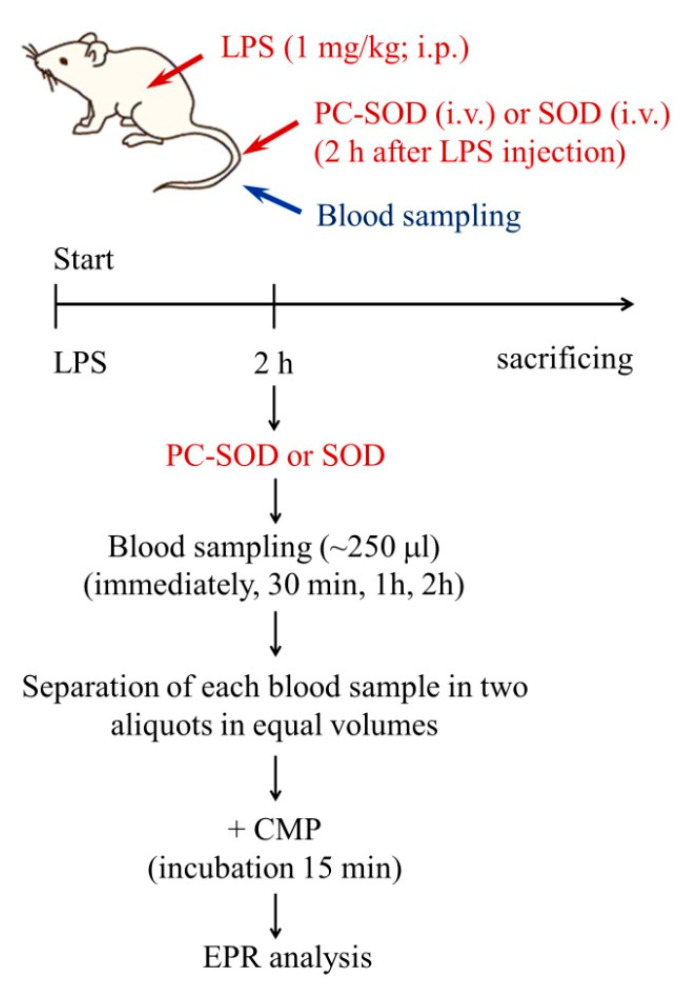
Schematic representation of treatment protocol. LPS was injected intraperitoneally (i.p.). Two hours later, the enzyme (SOD or PC-SOD) was injected intravenously (i.v.) through the tail vein. The blood samples were collected from the tail vein at four time intervals: immediately, 30 min, 1 h, and 2 h after the enzyme injection. Each blood sample was separated into two aliquots with equal volumes. CMP was added to each aliquot, incubated for 15 min, and analyzed by EPR spectroscopy. CMP—3-carbamoyl-2,2,5,5-tetramethylpyrrolidine-1-oxy; EPR—electron paramagnetic resonance; LPS—lipopolysaccharide; SOD—superoxide dismutase; and PC-SOD—lecithinized superoxide dismutase.

The experimental design is shown in Figure 4. Each mouse was anesthetized with 1.5% isoflurane using a face mask. Its body temperature was kept within 36 ± 1°C. Its tail vein was cannulated by a polyethylene tube (PE10, Intramedic, Becton Dickinson Co., Franklin Lakes, NJ, United States) connected to a syringe. All tubes and syringes were heparinized.

Two treatment protocols were used. In the first protocol, LPS (1 mg/kg b.w.) was injected. After two hours, SOD was injected in two doses: 1 mg/kg b.w. or 3 mg/kg b.w. The blood (~200–250 μL) was collected from the vein at three time intervals (immediately, 30 min, and 1 h) after enzyme injection. In the second protocol, LPS (1 mg/kg b.w.) was injected. After two hours, PC-SOD was injected at 3 mg/kg b.w. The blood (~200–250 μL) was collected from the vein at four time intervals (immediately, 30 min, 1 h, and 2 h) after enzyme injection. In both protocols, the maximum amount of blood collected from each mouse did not exceed 500 μL. Each blood sample was placed in an Eppendorf tube. Hematocrit was analyzed and controlled to be above 40% to avoid the presence of hemolysis during blood isolation. The presence of free transition-metal ions and free hemoglobin in the blood has been shown to interfere with the EPR signal due to their direct interaction with nitroxide [23,24]. Our previous experience with the quantification of free hemoglobin in blood samples isolated from mice indicated that hemolysis was negligible when the hematocrit was above 40–50%.

The nitroxide radical was initially dissolved in DMSO to prepare a 200 mM stock solution. Then, nitroxide (1 mM in buffer) was added to the isolated blood. The sample was placed in a glass capillary. The dynamic of the X-band EPR spectrum of the nitroxide probe in the sample was measured by EPR spectroscopy within 15 min under the following conditions: a microwave frequency of 9.4 GHz, a magnetic field strength of 336 mT, a microwave power of 2.0 mW, a field modulation frequency of 100 kHz, a field modulation amplitude of 0.063 mT, a time constant of 0.01 s, and a sweep width of 10 mT. The EPR signal intensity of the nitroxide probe (1 mM) in PBS was considered 100% and used as a control. The EPR spectrometer “Bruker ER 116 DS” was used in the analyses. The isolated samples were kept for a very short time in a closed system at room temperature before the measurements. The incubation time of CMP with blood was selected based on the kinetics of the EPR signal decay. The dynamics of the EPR signal of CMP in the blood were characterized by two phases: (i) a rapid and significant decrease within 15 min of incubation and (ii) a slow and insignificant decrease, with a plateau after that.

### 4.4. Statistical Analysis

All data are expressed as means ± standard deviation (SD). The normality of the distribution for all parameters of each experimental sample collected in each group was initially confirmed by using the Kolmogorov–Smirnov test. The most extreme differences for all experimental samples/groups were below the critical *D*-values. Based on the normality of the distribution in all samples/groups, the comparisons between them were performed using Student’s *t*-test for multiple comparisons. Two-tailed *p*-values of less than 0.05 were considered statistically significant.

## 5. Conclusions

The studies discussed above show that LPS induces the overproduction of ROS, leading to redox imbalance and oxidative stress in cells, tissues, and body fluids, which was confirmed using nitroxide radicals as redox-sensitive probes and EPR spectroscopy. SOD and its modified forms, such as PEG-SOD, abolish the effects of LPS on superoxide production. Our study partially confirmed the findings of other authors. To our knowledge, this is the first comparative EPR study demonstrating the effects of SOD and its modified form, PC-SOD, on the overproduction of superoxide in the blood of LPS-treated animals after i.v. injection of the enzymes. We established that PC-SOD suppresses oxidative stress in the bloodstream of LPS-treated mice, and its superoxide-scavenging and redox-modulating effects are more pronounced and last longer than those f unmodified SOD. We assume that this is due to the better stability and longer half-life of PC-SOD in the bloodstream, as well as its slower excretion due to its higher hydrophobicity compared with unmodified SOD. Indirect effects of PC-SOD, such as changes in metabolic pathways (e.g., mitochondrial respiration), inhibition of NOX, nitric oxide bioavailability, and others, may also interfere with LPS-triggered superoxide production in mice. However, these are only speculations that need experimental verification. Our study in mice with LPS-induced inflammation, as well as the experimental studies and clinical trials discussed, indicate that PC-SOD has translational importance. It is a promising pharmaceutical product for adjuvant therapy for various pathologies, which is well-grounded in a recent review article [13].

## Data Availability

The data presented in this study are available upon request from the corresponding author.

## Abbreviations

CMP—3-carbamoyl-2,2,5,5-tetramethylpyrrolidine-1-oxyl, EPR—electron paramagnetic resonance, LPS—lipopolysaccharide, MRI—magnetic resonance imaging, NOX—NADPH-dependent oxidase complex, PC-SOD—lecithinized superoxide dismutase, and SOD—superoxide dismutase.
